# Trends of limb amputation considering type, level, sex and age in Saskatchewan, Canada 2006–2019: an in-depth assessment

**DOI:** 10.1186/s13690-021-00759-1

**Published:** 2022-01-04

**Authors:** Samuel Kwaku Essien, David Kopriva, A. Gary Linassi, Audrey Zucker-Levin

**Affiliations:** 1 School of Rehabilitation Science, University of Saskatchewan, Health Science Building, E-Wing, Suite 3400, 3rd Floor, 104 Clinic Place, Saskatoon, SK S7N 2Z4 Canada; 2 Department of Surgery, University of Saskatchewan College of Medicine, Saskatoon, Canada; 3 Section of Vascular Surgery, Regina Qu’Appelle Health Region, University of Saskatchewan, Regina, Canada; 4 Department of Physical Medicine and Rehabilitation, University of Saskatchewan, Saskatoon, SK Canada

**Keywords:** Epidemiology, Incidence, Amputation, Lower extremity, Upper extremity

## Abstract

**Background:**

Most epidemiologic reports focus on lower extremity amputation (LEA) caused specifically by diabetes mellitus. However, narrowing scope disregards the impact of other causes and types of limb amputation (LA) diminishing the true incidence and societal burden. We explored the rates of LEA and upper extremity amputation (UEA) by level of amputation, sex and age over 14 years in Saskatchewan, Canada.

**Methods:**

We calculated the differential impact of amputation type (LEA or UEA) and level (major or minor) of LA using retrospective linked hospital discharge data and demographic characteristics of all LA performed in Saskatchewan and resident population between 2006 and 2019. Rates were calculated from total yearly cases per yearly Saskatchewan resident population. Joinpoint regression was employed to quantify annual percentage change (APC) and average annual percent change (AAPC). Negative binomial regression was performed to determine if LA rates differed over time based on sex and age.

**Results:**

Incidence of LEA (31.86 ± 2.85 per 100,000) predominated over UEA (5.84 ± 0.49 per 100,000) over the 14-year study period. The overall LEA rate did not change over the study period (AAPC -0.5 [95% CI − 3.8 to 3.0]) but fluctuations were identified. From 2008 to 2017 LEA rates increased (APC 3.15 [95% CI 1.1 to 5.2]) countered by two statistically insignificant periods of decline (2006–2008 and 2017–2019). From 2006 to 2019 the rate of minor LEA steadily increased (AAPC 3.9 [95% CI 2.4 to 5.4]) while major LEA decreased (AAPC -0.6 [95% CI − 2.1 to 5.4]). Fluctuations in the overall LEA rate nearly corresponded with fluctuations in major LEA with one period of rising rates from 2010 to 2017 (APC 4.2 [95% CI 0.9 to 7.6]) countered by two periods of decline 2006–2010 (APC -11.14 [95% CI − 16.4 to − 5.6]) and 2017–2019 (APC -19.49 [95% CI − 33.5 to − 2.5]). Overall UEA and minor UEA rates remained stable from 2006 to 2019 with too few major UEA performed for in-depth analysis. Males were twice as likely to undergo LA than females (RR = 2.2 [95% CI 1.99–2.51]) with no change in rate over the study period. Persons aged 50–74 years and 75+ years were respectively 5.9 (RR = 5.92 [95% Cl 5.39–6.51]) and 10.6 (RR = 10.58 [95% Cl 9.26–12.08]) times more likely to undergo LA than those aged 0–49 years. LA rate increased with increasing age over the study period.

**Conclusion:**

The rise in the rate of minor LEA with simultaneous decline in the rate of major LEA concomitant with the rise in age of patients experiencing LA may reflect a paradigm shift in the management of diseases that lead to LEA. Further, this shift may alter demand for orthotic versus prosthetic intervention. A more granular look into the data is warranted to determine if performing minor LA diminishes the need for major LA.

**Supplementary Information:**

The online version contains supplementary material available at 10.1186/s13690-021-00759-1.

## Background

Limb amputation (LA) is a life-changing procedure that impacts physical function and imposes economic burden on patients, caregivers, and the healthcare system [1–5]. Most epidemiologic reports focus on lower extremity amputation (LEA) caused by dysvascular disease, specifically diabetes mellitus (DM) [6, 7]. This focus is important to determine intervention efficacy and for predication purposes [8–10]; however, narrowing scope disregards the impact of other causes and types of LA diminishing the true incidence and societal burden.

Exploring LA incidence rates and trends over time can improve future tracking [11] and identify if a more rigorous investigation into inherent cause is needed [6, 12, 13]. Further, understanding trends in demographics of people living with LA can assist with identifying future orthotic, prosthetic and health service needs. For example, a rise in minor LA rate may demand more orthotic intervention such as custom insoles with toe fillers, while a rise in major LA may increase demand for prosthetic intervention. There are no previously published reports characterizing the entire cohort of people affected by LA in Saskatchewan, limiting comparison to other Provinces and countries. This study aimed to (1) assess the differential impact of lower and upper extremity amputation on LA incidence in Saskatchewan, and (2) explore overall amputation incidence rate by demographic profile in Saskatchewan and compare it with other Canadian trends.

## Methods

### Data sources

This study used hospital discharge data on all limb amputations (LA) over a 14-year period (2006–2019) performed in Saskatchewan and stipulated in the Canadian Classification of Health Interventions (CCI) codes (1SN93, 1SQ93, 1TA93, 1TK93, 1TM93, 1TV93, 1VA93, 1VC93, 1VG93, 1VQ93, 1UB93, 1UE93, 1UF93, 1UG93, 1UH93, 1UI93, 1UJ93, 1UK93, 1UM93, 1WA93, 1WE93, 1WI93, 1WJ93, 1WK93, 1WL93, 1WM93, 1WN93) (see supplementary file 1) [14]. This Discharge Abstract Database (DAD) is located at eHealth Saskatchewan accessible via Saskatchewan Health Quality Council. The DAD included all hospitals and health facilities in the province of Saskatchewan and contains up to 25 diagnoses and 20 interventions per hospitalization and is de-identifiably merged, by unique Saskatchewan personal health insurance numbers, to the Person Health Registration System. This enabled the extraction of matching demographic characteristics as stipulated in the provincial health insurance plan.

Subjects discharged for all causes of LA between January 1, 2006, and December 31, 2019 were included in the study. Type of amputation (upper extremity/lower extremity) were categorized by level as major (through/proximal to the ankle/wrist) or minor (distal to the ankle/wrist) based on CCI procedure codes [15]. If more than one LA was identified during the same hospitalization, the highest level LA was considered for analysis. If both UEA and LEA occurred at the same level (major or minor), the upper extremity was considered for analysis.

Demographics including age, sex, and admission date were retrieved. The databases have previously been used in population health-related research, and their validity, reliability, and completeness are established elsewhere [16–18]. Saskatchewan resident population from 2006 to 2019 by year, age groups (0–49 years, 50–74 years, and 75+ years) and sex (male/female), from the Saskatchewan Bureau of Statistics, was used as denominators to calculate rates [19].

### Data analysis

Saskatchewan’s annual LA rates for years 2006 to 2019 were calculated from total cases per yearly Saskatchewan resident population expressed per 100,000. To adjust for the effect of age and sex, a direct standardization method was performed using the 2011 population in Canada as the standard population [20, 21]. Rates were standardized by multiplying age group (0–49, 50–74, and 75 + years) and sex (male/female) specific rates by age and sex-specific weights. Then, a time-trend analysis was conducted to compare trends among population sub-groups.

Joinpoint regression [22] was then performed to identify and quantify significant changes in LA rates and by type (UEA/LEA) and level (major/minor). The regression model was fitted using a grid search method incorporated in the joinpoint software, assuming a constant variance and uncorrelated error term for the model [23]. The ideal number of breakpoints was determined via a permutation test with *p* < 0.05 considered statistically significant. Changes in each breakpoint and the full range of investigation (2006–2019) were reported as annual percent change (APC) and average annual percent change (AAPC) along with their respective 95% confidence intervals (95% CI).

A negative binomial regression was used to determine differences in temporal LA rate trends between males and females and among age groups (0–49 years, 50–74 years, 75+ years) [24]. An unadjusted model was fitted between the LA rate and each explanatory variable (age and sex) individually in two separate models (I and II). Then, the year of LA and interaction of year of LA by each explanatory variable (example, age by year and sex by year) were adjusted in each model. Relative rate (RR) and 95% CI were estimated. Differences in LA rate slopes between explanatory variables (e.g., female vs. male) over time were identified by an interaction *p* < 0.05.

## Results

### Cases

Over the 14 years (2006–2019), 5868 LA’s were performed in Saskatchewan, of which 4895 (83.4%) were LEA, and 973 (16.5%) were UEA. Minor LA predominated for both LEA (2777 minor, 2118 major) and UEA (884 minor, 89 major). More than one LA was identified in 47 hospitalizations eliminating 23 data points representing 0.4% of the entire study cohort over 14 years which insignificantly impacting our results.

### Trends in limb amputation by type (UEA/LEA)

**Fig. 1:**
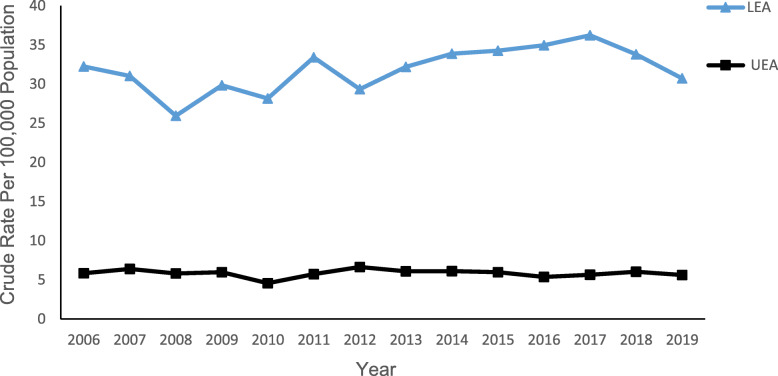
Crude rates of lower extremity amputation (LEA) and upper extremity amputation (UEA) in Saskatchewan, 2006–2019

Figure 1 illustrates a comparison of LEA and UEA trends from 2006 to 2019. Consistently higher rates of LEA (31.86 ± 2.85 per 100,000) than UEA (5.84 ± 0.49 per 100, 000) were observed. The Joinpoint analysis (Table 1) revealed both LEA and UEA insignificantly declined of 0.5% (AAPC -0.5 [95% CI − 3.8 to 3.0]) and 0.2% (AAPC -0.2 [95% CI − 1.5 to1.2]) respectively over the entire study period. Further analysis revealed LEA rates significantly increased 3.15% (APC 3.15 [95% CI 1.1 to 5.2]) (*p* < 0.05) from 2008 to 2017 countered by two insignificant periods of decline (2006–2008 and 2017–2019). In contrast, no break points were identified in the rate of UEA during the study period.

**Table 1:** Annual Percent Change in lower and upper extremity amputation rate, 2006–2019

Amputation type/ level	Breakpoints	APC (95% CI)	AAPC (95% CI)
Lower Extremity	2006–2008	−8.24 (− 23.6 to 10.3)	− 0.5 (− 3.8 to 3.0)
	2008–2017	3.15* (1.1 to 5.2)	
	2017–2019	−8.02 (−23.5 to 10.5)	
Upper Extremity	2006–2019	−0.16 (−1.5 to 1.2)	− 0.2 (− 1.5 to 1.2)
Upper Extremity Minor	2006–2019	−0.62 (−2.1 to 0.9)	−0.6 (− 2.1 to 0.9)
Lower Extremity Minor	2006–2019	3.88* (2.4 to 5.4)	3.9* (2.4 to 5.4)
Lower Extremity Major	2006–2010	−11.14* (− 16.4 to −5.6)	−4.6* (−7.6 to − 1.6)
	2010–2017	4.20* (0.9 to 7.6)	
	2017–2019	−19.49* (−33.5 to −2.5)	

*APC* Annual Percent Change, *AAPC* Average Annual Percent Change, *Cl* Confidence Interval

*Indicates a statistically significant breakpoint *p* < 0.05

### Trends in limb amputation by level (major/minor)

**Fig. 2:**
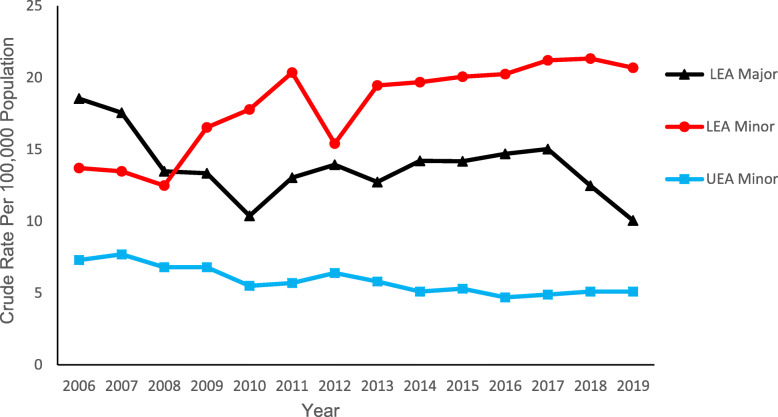
Crude rates of lower extremity amputation (LEA) in Saskatchewan stratified by calendar year, level, and type of amputation

Figure 2 depicts type (LEA/UEA) and level (major/minor) of LA trends between 2006 and 2019. The rate of minor UEA was lower than the rate of minor LEA (5.26 ± 0.55 and 18.03 ± 3.12 per 100,000 population, respectively; *p* < 0.001) with limited major UEA sample size precluding comparison. The 14-year rate of major LEA was less than the rate of minor LEA (13.83 ± 2.30; 18.03 ± 3.12 respectively; *p* < 0.001). However, the rate of major LEA exceeded that of minor LEA in years 2006–2008 with reversal in 2009 and persistence through the remainder of the study period.

Joinpoint analysis (Table 1) revealed minor LEA rates increased 3.9% over the study period (AAPC 3.9 [95% CI 2.4 to 5.4]) (*p* < 0.05) with no breakpoints while major LEA rates decreased 4.6% (AAPC -4.6 [95% CI − 7.6 to − 1.6]) with three break points. From 2010 to 2017 major LEA rates increased 4.2% (APC 4.2 [95% CI 0.9 to 7.6]) (*p* < 0.05) countered by a decrease of 11.14% in 2006–2010 (APC -11.14 [95% CI − 16.4 to − 5.6]) (*p* < 0.05) and 19.5% in 2017–2019 (APC -19.49 [95% CI − 33.5 to − 2.5]) (*p* < 0.05). Minor UEA rates insignificantly decreased (APC -0.62 [95% CI -2.1-0.9]) over the entire study period (2006 and 2019) with no breakpoints identified. Too few major UEA occurred to warrant analysis.

### Trend in limb amputation by age

**Fig. 3:**
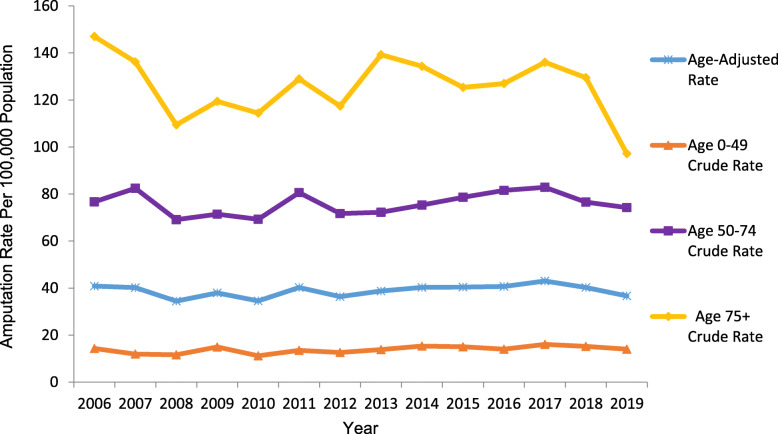
Age-adjusted and age-stratified crude amputation rates by year

LA rates were higher in those aged 75+ years compared to those aged 50–74 and 0–49 years during the entire study period 2006 to 2019 (Fig. 3).

The unadjusted results in Table 2 revealed that people aged 50–74 years and 75+ years were respectively 5.49 (95% Cl 5.08 to 5.93) and 9.10 (95% Cl 8.45 to 9.80) times more likely to experience LA compared to those aged 0–49 years. After adjusting for the year of amputation and the interaction between age and year of amputation, the RR increased to 5.92 (95% Cl 5.39 to 6.51) and 10.58 (95% Cl 9.26 to 12.08). In addition, the statistically significant interaction between age and year of amputation (*p* = 0.007) indicates the changes in amputation rate over the 14 years were different among the three age group with an increase in the rate of LA increasing with age.

**Table 2:** Unadjusted and adjusted relative rates for amputations among population groups

Variables	Unadjusted				Adjusted			
	Coef.	RR	95%Cl	*P*-value	Coef.	RR	95%Cl	*P*-value
**Model I**								
**Age/years**								
0–49		1.00				1.00		
50–74	1.703	5.49	(5.08–5.93)	< 0.001	1.779	5.92	(5.39–6.51)	< 0.001
75+	2.209	9.10	(8.45–9.80)	< 0.001	2.359	10.58	(9.26–12.08)	< 0.001
**Year**	−0.001			0.938	0.024			0.012
Age and Year Int*					−0.010			0.007
**Model II**								
**Sex**								
Female		1.00				1.00		
Male	0.886	2.43	(2.30–2.56)	< 0.001	0.806	2.24	(1.99–2.51)	< 0.001
**Year**	0.005			0.565	−0.013			0.271
Sex and Year Int*					0.011			0.120

*RR* Relative Rate, *Cl* Confidence Interval, Int* Interaction

### Trends in limb amputation by sex

**Fig. 4:**
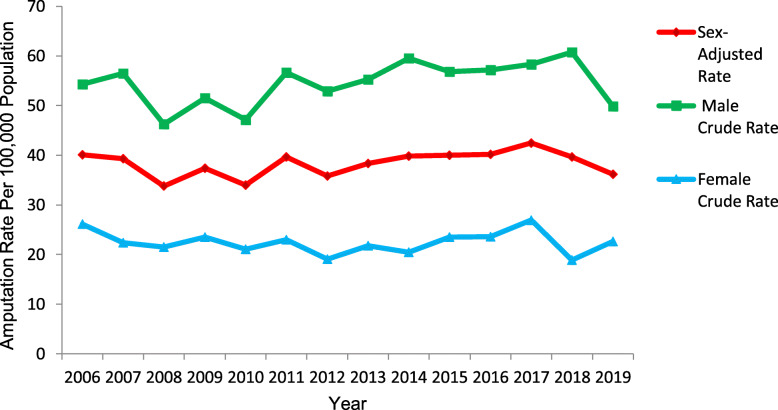
Sex-adjusted and sex-stratified crude amputation rates by year

For the 14-year study period (2006–2019) males had significantly higher rates of LA than females (*p* < 0.001) (Fig. 4). The sex-stratified crude rates are consistent with the associations found via the negative binominal regression in Table 2.

The unadjusted model identified males were 2.43 (95% Cl 2.30 to 2.56) times more likely to undergo LA than females. After adjusting for the year of amputation and sex-by-year of amputation interaction, the RR decreased to 2.24 (95% Cl 1.99 to 2.51) however, the sex-by-year of amputation interaction was not statistically significant (*p* = 0.120), suggesting LA rate did not differ over time by sex.

### Age-and-sex adjusted rates

The average age- and sex-adjusted LA rates from 2006 to 2019 were 38.91 and 38.35 per 100,000 population, respectively. Despite the variable trends observed in both crude age- and –sex stratified rates over the study period, after controlling for age and sex, the age- and -sex adjusted rates remained stable especially between 2012 and 2016–2017, although there were fluctuations in the crude rates over the study period. The decline observed in the age-adjusted rate from 2017 to 2019 could be attributed to the change in the population base (more older people in 2017–2019) of those aged 50+ years.

## Discussion

This is the first study to report changes in limb amputation rates considering all amputations by type, level, sex and age in Saskatchewan from 2006 to 2019 irrespective of cause of amputation. We chose to do this because limiting the cohort to the most prevalent cause and level of amputation limits the understanding of societal impact. We found the overall rate of LA (both UEA and LEA) remained stable over the 14-year study period however fluctuations were identified in the rate of LEA during the study period. LEA significantly increased 3.15% (APC 3.15 [95% CI 1.1 to 5.2]) (*p* < 0.05) from 2008 to 2017 but this was countered by two periods of insignificant but impactful decline in LEA rate (2006–2008 and 2017–2019).

An earlier published Canadian study found declining trends in LEA rates at national and provincial levels over a 6 year period between 2006 and 2011 [6]. We performed a joinpont analysis on Imam et al.’s published age-adjusted rate data and found that Canada’s national LEA rate declined by 1.6%, Manitoba declined by 0.3%, Nova Scotia declined by 1.3%, Ontario declined by 2.1%, Newfoundland declined by 3.9%, and Saskatchewan declined by 2.4% [6]. In contrast, a 1.3% increase in the rate of LEA was identified in Alberta, a province with a similar sociodemographic profile as Saskatchewan during the same time interval [6]. Our study identified that expanding the data set to 2019 alters this trend revealing an insignificant (0.5%) LEA rate of decline.

Our findings were also compared globally. Li et al. (2012) reported a decrease in the discharge rate of nontraumatic LEA performed in the United States from 11.2 per 1000 persons with diabetes in 1996 to 3.9 in 2008 while the rate of LEA did not change in persons without diabetes [25]. Geiss et al. (2019) also reported a significant decrease in the hospitalization rate of nontraumatic LEA performed in the United States from 5.38 per 1000 adults with diabetes in 2000 to 3.07 in 2009 but this decline rebounded to 4.62 in 2015 [26]. Unlike Li (2012), Geiss et al. found a decline in nontraumatic LEA in adults without diabetes from 0.23 per 1000 in 2000 to 0.18 in 2015 [26]. The reported trends illustrate the importance of examining LA data over long periods of time and the need for further analyses to identify external factors influential to these rates.

The low incidence of UEA found in our cohort was expected and indicates trends in the overall LA rate are largely due to the influence of LEA. This imbalance is in line with other studies worldwide [27, 28] and mainly due to differences in the leading causes of LEA and UEA [6, 29, 30]. Vascular disease, specifically diabetes is the leading cause of LEA [31] responsible for more than 65% of LEA in Canada from 2006 to 2011 [6] while trauma accounts for 80–90% of all UEA [29, 30]. Our study cohort, specific data reported elsewhere, had a similar causal distribution with diabetes identified as the predominant (54.1–81.5%) predisposing factor, followed by peripheral vascular disease not associated with diabetes (4.2–16.3%) and then trauma (4.3–8.7%) [32].

Further analysis of type of amputation revealed most UEA were minor (distal to the wrist) with major (through and proximal to the wrist) UEA rates not assessed because of limited sample size. The predominance of minor UEA is supported by Ziegler-Graham’s who reported minor UEA accounted for 92% of the 541,000 UEA performed in the US in 2005 [29].

Like UEA, most LEA were minor (distal to the ankle). Our finding that LEA insignificantly declined (AAPC -0.5 [95% CI − 3.8 to 3.0]) from 2006 to 2019 was disappointing but we were encouraged by the 3.9% (AAPC 3.9 [95% CI 2.4 to 5.4]) (*p* < 0.05) increase in minor LEA associated with a decline of 4.6% (AAPC -4.6 [95% CI − 7.5 to − 1.6]) (*p* < 0.05) in major (through or proximal to the ankle) LEA rate over the study period. Fluctuations in major LEA rate nearly corresponded with fluctuations in the overall LEA rate with one period of rising rates from 2010 to 2017 (APC 4.2 [95% CI 0.9 to 7.6]) (*p* < 0.05) countered by two periods of decline 2006–2010 (APC -11.14 [95% CI − 16.4 to − 5.6]) (*p* < 0.05) and 2017–2019 (APC -19.49 [95% CI − 33.5 to − 2.5]) (*p* < 0.05). Our findings are supported by other studies that report increased rates of minor LEA over the past two decades [26, 33–35]. Geiss et al. (2019) identified a 62% increase in the rate of nontraumatic minor LEA but, in contrast to our findings, they reported a 29% increase in nontraumatic major LEA between 2009 and 2015 [26]. The increase in minor LEA rates may be reflective a paradigm shift in the surgical care of people with foot ulcers with minor, often repeated LEA performed in efforts to save the limb and maintain a functional foot on which to walk [36]. The increase in minor LEA may shift the need to orthotic intervention such as foot plates and toe fillers, as opposed to prosthetic intervention after LA [37].

Our finding that males were twice as likely to undergo LA than females (RR = 2.24 [95% CI 1.99 to 2.51]) is consistent with the literature [6, 25, 26, 38]. The higher rate of LA among men, as evidenced by the current study’s crude rate estimates was consistent with Canada’s national amputation rate pattern for 2006–2011 [6]. Internationally, a study examining German national discharge abstract data found a significant decrease in the rate of LLA for both men and women from 2005 to 2015, with men experiencing less of a decrease (− 2.6%) than women (− 25%) [38]. This decline in overall LLA rate was attributed more higher-level initial amputations, thus decreasing the need for revision preocedures often associated with minor amputation [38].

Our finding that older persons are disproportionately affected by LA is also consistent with the literature [39]. We found persons aged 50–74 years and 75+ years were respectively 5.9 (RR = 5.92 [95% Cl 5.39 to 6.51]) and 10.6 (RR = 10.58 [95% Cl 9.26 to 12.08]) times more likely to undergo LA than those aged 0–49 years (*p* < 0.001). We also found the rate of LA increased for persons 50+ years of age over the study period.

Aziz et al. found age 60+ years to be a significant predictor of amputation in people with diabetic foot infections [39]. Two diverse statistical methods applied to the current study data showed that age, especially those aged 50–74 years and 75+ years was a stronger amputation rate driver. This could in part be attributed to accelerated risk of developing vascular complications as people live longer with chronic vascualar diseases, such as diabetes [13, 40]. Since diabetes was the major predictive factor for LA in our cohort [34] our findings give rise to the well documented need for pro-active diabetes care. Data from Diabetes Canada [41] and the Government of Canada-Action for Seniors Report [42] contribute to future concerns. Diabetes Canada reports that in Saskatchewan, in 2018, the largest group of persons with diabetes were 60–74 years of age, followed by persons 75+ years of age [41]. The prevalence of diabetes is expected to increase from 2015 reported 9.3 to 12.1% of the population by 2025 [43]. Further, the government of Canada predicts a rise in the percentage of older Canadians, with life expectancy for both men and women also predicted to rise [42]. Collectively, these data will likely shift the currently declining trend in amputation rate in Saskatchewan.

## Strengths and limitations

This study used diverse statistical methods to thoroughly assess and elucidate the influence of type, level, age and sex on amputation rate in the province. The study was limited by UEA inadequate samples.

## Conclusion

Our broader examination of the epidemiology of LA, independent of cause, identified that rate of LA (both UEA and LEA) remained stable over the 14-year study period in Saskatchewan, Canada. Not surprising were the findings that the rate of male LA more than twice outnumbered that of female, that LEA thrice outnumbered UEA and that LA increased with increasing age. Interesting was the finding that overall LEA rate was largely driven by the rate of major LEA. In summary, we found a declining rate of major LEA concomitant with an increasing rate of minor LEA with a rising age of persons affected by LA in Saskatchewan from 2006 to 2019. Further research is needed to determine if these findings are due to medical and/or social interventions adopted over the past two decades to counter complications of vascular disease and to determine if changing major and minor LA ratios alters orthotic and prosthetic prescription.

## Supplementary Information

**Additional file 1.**

## Data Availability

The datasets used and/or analysed during the current study are available from the corresponding author on reasonable request.

## Abbreviations

LA: Limb amputation
DM: Diabetes mellitus
DAD: Discharge abstract disease
APC: Annual percent change
AAPC: Average annual percent change
CI: Confidence interval
RR: Relative rate
LEA: Lower extremity amputation
UEA: Upper extremity amputation
CCI: Canadian Classification of Health Intervention
