# Supplementary material for: Trends of limb amputation considering type, level, sex and age in Saskatchewan, Canada 2006–2019: an in-depth assessment
Source: Arch Public Health. 2022 Jan 4;80:10. doi: 10.1186/s13690-021-00759-1 (PMC8729075; doi:10.1186/s13690-021-00759-1)
Supplement: Supplementary file 1 — Additional file 1. [file 13690_2021_759_MOESM1_ESM.pdf]

Supplementary file 1 of exact representation and details of each CCI code

CCI CODES

Upper extremity amputations

Major

- 1SN93 = Amputation at scapula
- 1TA93 = Amputation at shoulder joint
- 1TK93 = Amputation at humerus
- 1TM93 = Amputation at elbow joint
- 1TV93 = Amputation at radius and ulna
- 1UB93 = Amputation at wrist joint

Minor

- 1UE93 = Amputation at first metacarpal bone
- 1UF93 = Amputation at other metacarpal bones
- 1UG93 = Amputation at other metacarpophalangeal joint(s)
- 1UH93 = Amputation at first metacarpophalangeal joint
- 1UI93 = Amputation at first phalanx of hand
- 1UJ93 = Amputation at other phalanx of hand
- 1UK93 = Amputation at other interphalangeal joint of hand
- 1UM93 = Amputation at first interphalangeal joint of hand

Lower extremity amputations

Major

- 1SQ93 = Amputation at pelvis
- 1VA93 = Amputation at hip joint
- 1VC93 = Amputation at femur
- 1VG93 = Amputation at knee joint
- 1VQ93 = Amputation at tibia and fibula
- 1WA93 = Amputation at ankle joint

Minor

- 1WE93 = Amputation at tarsal bones and intertarsal joints (hindfoot, midfoot)
- IWI93 = Amputation at first metatarsal bone and first metatarsophalangeal joint
- 1WJ93 = Amputation at tarsometatarsal joints, other metatarsal bones and other metatarsophalangeal joints (i.e., forefoot)
- 1WK93 = Amputation at first phalanx of foot
- 1WL93 = Amputation at phalanx of other foot
- 1WM93 = Amputation at other interphalangeal joints of toe
- 1WN93 = Amputation at first interphalangeal joint of toe
